# A Novel Packaged Ultra-High Q Silicon MEMS Butterfly Vibratory Gyroscope

**DOI:** 10.3390/mi13111967

**Published:** 2022-11-13

**Authors:** Lu Jia, Guowei Han, Zhenyu Wei, Chaowei Si, Jin Ning, Fuhua Yang, Weihua Han

**Affiliations:** 1 Engineering Research Center for Semiconductor Integrated Technology, Institute of Semiconductors, Chinese Academy of Sciences, Beijing 100083, China; jialu@semi.ac.cn (L.J.); zywei97@semi.ac.cn (Z.W.); schw@semi.ac.cn (C.S.); ningjin@semi.ac.cn (J.N.); fhyang@semi.ac.cn (F.Y.); 2 Center of Materials Science and Optoelectronics Engineering, University of Chinese Academy of Sciences, Beijing 100049, China; 3 School of Electronic, Electrical and Communication Engineering, University of Chinese Academy of Sciences, Beijing 100049, China; 4 State Key Laboratory of Transducer Technology, Chinese Academy of Sciences, Beijing 100083, China

**Keywords:** butterfly vibratory gyroscope, wafer-level sandwich packaging, ultra-high Q factor

## Abstract

A novel three-dimensional (3D) wafer-level sandwich packaging technology is here applied in the dual mass MEMS butterfly vibratory gyroscope (BFVG) to achieve ultra-high Q factor. A GIS (glass in silicon) composite substrate with glass as the main body and low-resistance silicon column as the vertical lead is processed by glass reflow technology, which effectively avoids air leakage caused by thermal stress mismatch. Sputter getter material is used on the glass cap to further improve the vacuum degree. The Silicon-On-Insulator (SOI) gyroscope structure is sandwiched between the composite substrate and glass cap to realize vertical electrical interconnection by high-vacuum anodic bonding. The Q factors of drive and sense modes in BFVG measured by the self-developed double closed-loop circuit system are significantly improved to 8.628 times and 2.779 times higher than those of the traditional ceramic shell package. The experimental results of the processed gyroscope also demonstrate a high resolution of 0.1°/s, the scale factor of 1.302 mV/(°/s), and nonlinearity of 558 ppm in the full-scale range of ±1800°/s. By calculating the Allen variance, we obtained the angular random walk (ARW) of 1.281°/√h and low bias instability (BI) of 9.789°/h. The process error makes the actual drive and sense frequency of the gyroscope deviate by 8.989% and 5.367% compared with the simulation.

## 1. Introduction

The automotive industry, military equipment, consumer electronics and other application markets require MEMS gyroscopes for lower cost, easier integration, higher sensitivity, lower vibration sensitivity, higher Q factor and better reliability [1,2,3,4]. Numerous scientific research institutions have put a lot of efforts into structure design, micromachining technology and readout electronic circuits. G. Andersson, who worked at IMEGO Institute, first proposed the butterfly gyroscope in 1999 [5]. After continuous development and progress, this structure shows the ability to achieve high performance [3,4,6,7].

Most MEMS gyroscopes based on surface micromachining have a flat structure [8] and low sensitivity [1,7]. Comb capacitors are used to excite the structure and detect the output [9]. The processing of combs is particularly complex and an uneven electrode gap will lead to excessive ZRO [1,9]. BFVG works based on plate electrodes, which avoids the manufacture of combs [3]. This simple structure design does not have many suspension beams, anchors and combs, and the sense vibration occurs outside the plane with higher sensitivity. From structural design to processing, the butterfly gyroscope has unique advantages [2]. 

The Q factor is defined as the ratio of stored energy to dissipated energy in each vibration cycle. Increasing the Q factor can improve the performance of the gyroscope, such as signal-to-noise ratio, resolution, power consumption and bias instability (BI) [10,11]. Appropriate packaging technology can protect sensitive and fragile structures from external impact, and thus obtain a high Q factor. In this case, the combination of three-dimensional (3D) wafer-level packaging technology with anodic bonding and vertical interconnection technology has great potential in realizing high-performance BFVG [12,13,14,15]. At present, in the bonding package process of inertial MEMS devices, the composite substrate is mostly glass or silicon perforated, and the vertical wire is mostly made of electroplating metal. So the composite substrate contains at least two materials, metal in silicon or metal in the glass. The composite substrate will give rise to thermal stress mismatch due to the different thermal expansion coefficients of the two materials. It will cause air leakage in the packaging chamber [16,17]. This is one of the main reasons for the failure of bonding packages [18,19].

In this work, a new 3D wafer-level sandwich packaging technology was used for the BFVG structure. Firstly, we designed a dual mass butterfly gyroscope. By etching grooves under the silicon structural layer, the vertical electrostatic force can bend the asymmetrical beam in the vertical and horizontal directions. Secondly, to solve the above problem of bonding package failure, a composite substrate with glass as the main body and a low-resistance silicon column as the vertical lead is processed by glass reflow technology. Getter material is sputtered on the glass cap to further improve the vacuum. The sensor structure is processed on the SOI wafer. The three-layer structure enables vertical interconnection and high-vacuum packaging through anodic bonding. Finally, the BFVG was tested and characterized by the self-developed control circuit system.

## 2. Structural Design and Operation Principle

**Figure 1:**
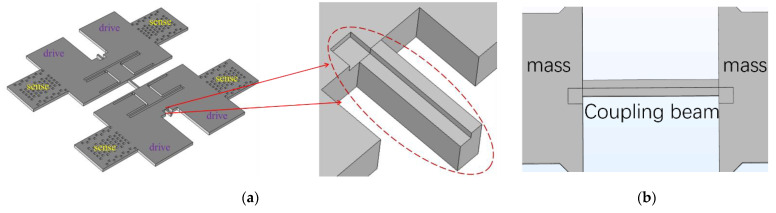
(**a**) A schematic illustration of the BFVG structure; (**b**) back of coupling beam.

The structure diagram of the designed BFVG is shown in the left part of Figure 1a. The sensing element consists of two proof masses, a coupling beam and folding beams. Two proof masses are connected with each other through the coupling beam, and are symmetrically distributed at the anchors. Because its shape is similar to a butterfly, this type of structure is called a butterfly vibratory gyroscope (BFVG). The coupling beam is one of the most critical components in the BFVG, as shown in the Figure 1b. By wet etching and forming DRIE grooves under the silicon structure layer, the vertical electrostatic force can bend the asymmetrical beam in the vertical and horizontal directions. The dimension parameters of the coupling beam and other structural parts are shown in Table 1.

The basic operating principles of the BFVG are as follows. The drive voltage excites the structure to vibrate; if there is an external angular velocity input, the gyro will oscillate by coriolis force in the sense direction. In this structure, the movement of the mass on the Z-axis causes gap change, and the sense electrode detects the coriolis effect by measuring the capacitance change caused by the gap change.

The multi-physical field simulation of the designed BFVG structure is carried out by using COMSOL multi-physics software. Table 1 shows the important structure parameters in the simulation. 

**Figure 2:**
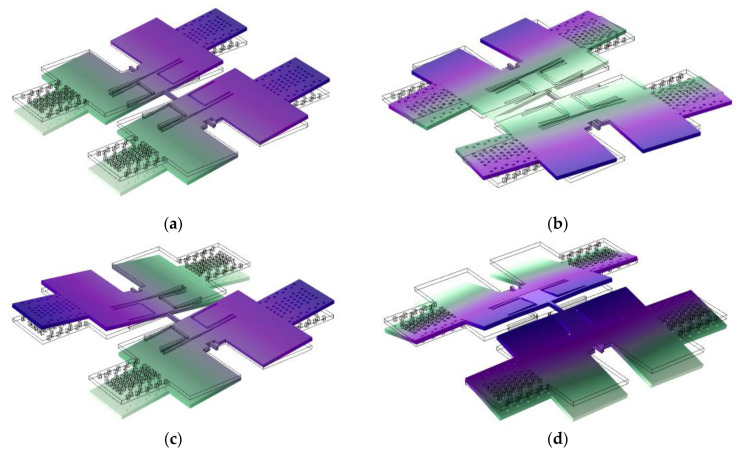
The modal simulated results of BFVG: (**a**) mode 1; (**b**) mode 2; (**c**) mode 3; (**d**) mode 4.

As shown in Figure 2, the first four modes of the designed BFVG are obtained by modal simulation. Figure 2a mode 1: the coupling beam is twisted, and the two proof masses oscillate in phase around the horizontal axis. Figure 2b mode 2: the coupling beam is bent horizontally, and the two proof masses oscillate out of phase around an almost vertical axis. Figure 2c mode 3: the coupling beam is twisted, and the two proof masses oscillate out of phase around the horizontal axis. Figure 2d mode 4: the coupling beam is vertically bent, and the two proof masses oscillate out of phase around an almost vertical axis. The second and third vibration modes are selected as the drive and sense modes of the gyroscope, respectively. These two modes of anti-phase operation can greatly reduce the sensitivity of the structure to external linear and angular vibration.

**Figure 3:**
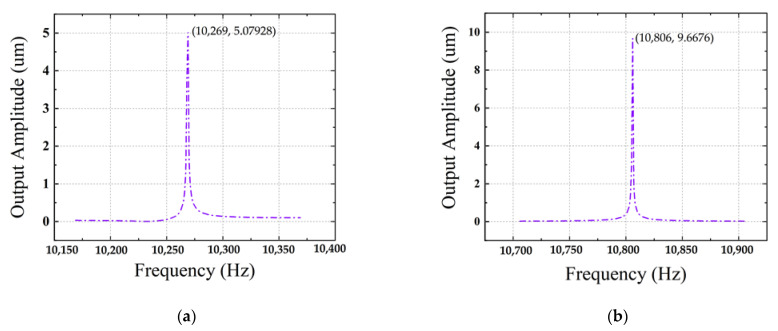
(**a**) Drive mode frequency domain. (**b**) Sense mode frequency domain.

The amplitude frequency response of drive mode and sense mode obtained by COMSOL frequency scanning are shown in Figure 3. The theoretical drive frequency and sense frequency of the device are 10,269 Hz and 10,806 Hz. The frequency difference between these two modes is within 1 kHz. Under the condition of ensuring the bandwidth, reducing the frequency mismatch as much as possible can obtain a greater response.

## 3. Fabrication of BFVG

Encapsulation is crucial for MEMS sensors. It can not only protect the movable structure from the influence of the external environment, but also realize the transmission of electrical signals.

Previous butterfly gyroscopes in this group are packaged with traditional ceramic shells. This is a chip-level packaging technology, which is often used in the packaging of integrated circuits and MEMS devices. After the completion of the wafer fabrication process, the diced chip is put into the ceramic shell for packaging. However, this packaging method has the problems of a large size and high costs, and it cannot be mass- produced [20]. A novel 3D sandwich structure wafer-level package technology is studied and applied to the designed BFVG.

### 3.1. Novel Manufacturing Technology

**Figure 4:**
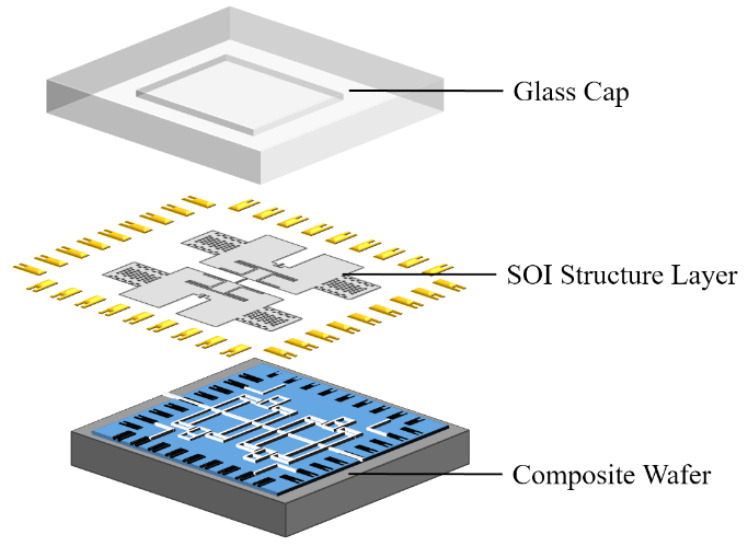
3D “sandwich structure” wafer-level-packaged BFVG.

As shown in Figure 4, the butterfly gyroscope in a 3D wafer-level package adopts a sandwich structure with a glass cap–SOI structure layer–composite substrate from top to bottom. There are many advantages to the new manufacture technology.

(1) The glass cap is dug to form a cavity as the movable space of the sensor structure. Getter material is sputtered in the cavity to improve the vacuum degree. 

(2) The thickness of the top silicon is 20 μm , which is the main sense structure area. The buried oxygen layer can quickly form a movable sense structure through HF gas release. 

(3) The composite substrate is processed by the silicon in glass (SIG) reflow process and through silicon vias (TSV) technology. Silicon pillars form electrically isolated electrodes due to the insertion of glass. The three layers of glass cap–SOI structure layer–composite substrate form a seal through anodic bonding. At the same time, the vertical electrical interconnection is realized through the silicon pillars, which reduces the interconnection length and the power consumption and delays the transmitting of electrical signals.

(4) A multilayer structure requires multiple bonding. Compared with silicon–silicon bonding and gold–silicon bonding, silicon–glass anodic bonding has certain advantages in terms of cost, reliability and efficiency. At room temperature (300 K), the coefficient of the thermal expansion (CTE) of silicon is about $2.59\times 10^{-6}\;K^{-1}$ , and the CTE of the selected borosilicate glass (Pyrex 7740) is about $3.25\times 10^{-6}\;K^{-1}$ . The thermal expansion coefficient of aluminum is about $23\times 10^{-6}\;K^{-1}$ . In comparison, the thermal expansion coefficients of borosilicate glass (Pyrex 7740) and silicon are very close. This makes the residual stress between Pyrex 7740 glass and silicon wafer smaller after anodic bonding.

### 3.2. Fabrication Procedure

**Figure 5:**
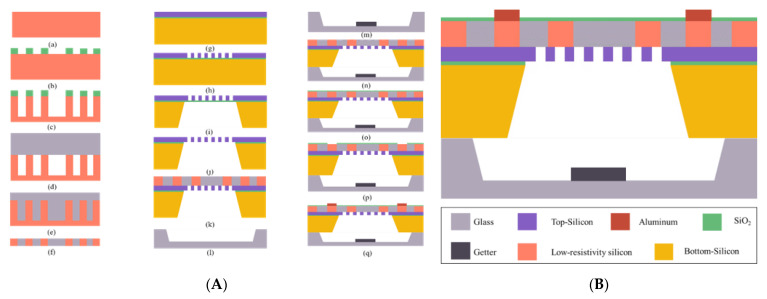
(**A**) The fabrication process of BFVG in 3D wafer-level packaging. (**B**) The device structure of the sensing element.

(1) Processing of composite substrate: Figure 5(Aa) Select silicon wafer—A 4-inch p-type <100> crystalline silicon wafer with low resistivity (0.0009 Ω·cm ) is used to realize low loss electrical interconnection. Figure 5(Ab) Lithography—A pattern is formed on the photoresist, and the photoresist and oxide layer are used as masks for the next step of deep silicon etching. Figure 5(Ac) DRIE—The deep reactive ion etching process is carried out to etch the silicon wafer to form a mold with a certain depth of grooves for the glass reflow process. Figure 5(Ad) Anodic bonding—Under a $10^{-2}$ mbar vacuum condition at 330 °C, silicon substrate with the same thickness is bonded with Pyrex glass. Figure 5(Ae) Glass reflow—We put the bonding wafer into a high-temperature furnace. When the temperature rises to more than 750 °C, the molten glass begins to be pushed into the silicon groove under the pressure difference between inside and outside. After the temperature reaches 1050 °C, it is held for 2 h to ensure that the glass is completely filled. Figure 5(Af) CMP—the composite substrate forces silicon vertically through holes through double-sided chemical mechanical polishing, and this provides a good surface quality for subsequent process steps. 

(2) Processing of structural layer: Figure 5(Ag) Select SOI chip—A 4-inch n-type <100> crystalline silicon chip is used, with bottom silicon thickness of 300 μm , buried oxygen layer thickness of 1 μm , and top silicon thickness of 20 μm . Figure 5(Ah) ICP—The top silicon layer is used as the sense layer, and the silicon oxide is used as the mask to etch the silicon sense structure and electrodes. Figure 5(Ai) Wet etching—We etch the bottom silicon to expose the area to be released. Figure 5(Aj) HF gas release—the HF gas releases the ${\mathrm{SiO}}_{2}$ area, allowing the sense structure to be movable. Figure 5(Ak) Anodic bonding—the SOI wafer (the surface of the top silicon layer) is anodically bonded with the composite substrate. 

(3) Glass cap processing: Figure 5(Al) Wet etching—HF/HCl/DI water is used to partially wet etch the Pyrex glass to form a cavity. Figure 5(Am) Sputtering—A highly porous Zr-based getter material is sputtered into the glass cavity. This getter material can absorb $H_{2}O$ , $H_{2}$ , ${\mathrm{CO}}_{2}$ , $N_{2}$ and other gases in the cavity after activation, and improve the vacuum degree. Figure 5(An) Anodic bonding—The glass cap and SOI wafer (the surface of the bottom silicon layer) are anodically bonded under a pressure of about $10^{-5}$ mbar. 

(4) Preparation of electrodes: Figure 5(Ao) PECVD—We deposit silicon dioxide on the surface of the composite substrate as an insulating layer. Figure 5(Ap) Etching—A photoresist is used as a mask to etch the electrode area in the silicon dioxide layer. Figure 5(Aq) Electrode—We deposit aluminum and locally wet etch to form electrodes on the surface of the composite substrate. 

**Figure 6:**
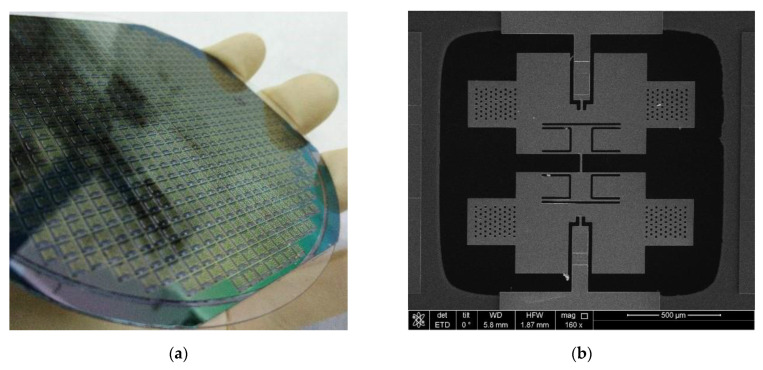

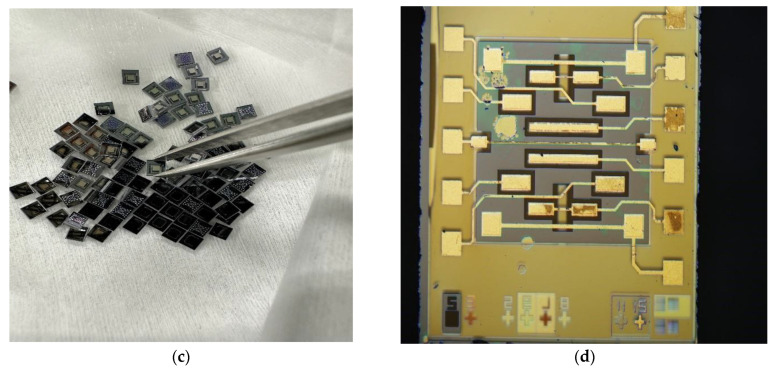
(**a**) Picture of composite wafer. (**b**) SEM image of the MEMS structure. (**c**) Photograph of wafer-level package MEMS butterfly vibratory gyroscope. (**d**) Microscopic image of a single packaged gyroscope.

Figure 6a shows a photo of the processed composite wafer. To observe the single BFVG clearly, the whole structure was observed with a scanning electron microscope (SEM). As shown in Figure 6b, the gyro structure is completely flat, smooth, free of stains, and has good morphology.

## 4. Characterization and Experimental Results 

**Figure 7:**
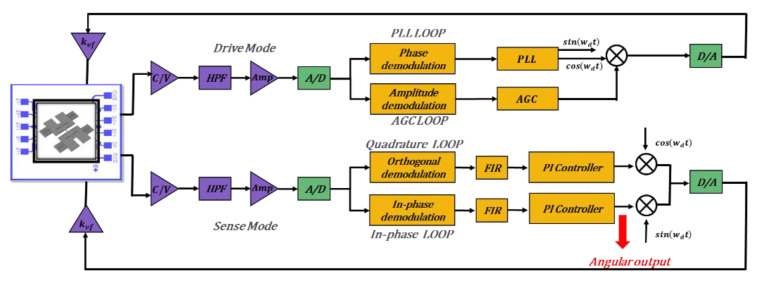
A schematic depiction of the control circuit system for BFVG.

In order to further characterize the reliability of the 3D wafer-level packaging technology and the performance of the designed butterfly gyroscope structure, BFVG is installed on a special shell and connected with the self-developed control circuit. As shown in Figure 7, the gyro signal is mainly controlled by two closed-loop circuits. Each loop includes a front-end analog circuit (purple), a digital control circuit (yellow) and a conversion circuit (ADC, DAC). The drive signal is transmitted to a 14 bit analog-to-digital converter (ADC) with a sampling rate of 200 kHz after CV conversion and high-pass filter amplification in the front-end analog circuit. The digital signal output by ADC is processed by the FPGA board (Spartan-6, XC65LX45). The digital control circuit mainly includes phase-locked loop (PLL) and automatic gain control (AGC), which are used to stabilize the frequency and amplitude of the drive signal of the gyroscope. These guarantee the stable operation of the gyroscope. The signal output from the FPGA board is returned to the gyro through the DAC to form a closed loop. The sense signal is also transmitted to the FPGA board through the front-end analog circuit and ADC. The difference is that the sense signal is detected and collected by the force rebalance (FTR) and quadrature error correction control system in the digital circuit. Similarly, the signal output from the FPGA board is returned to the gyroscope through the DAC to form a closed loop.

### 4.1. Frequency Characteristic

**Figure 8:**
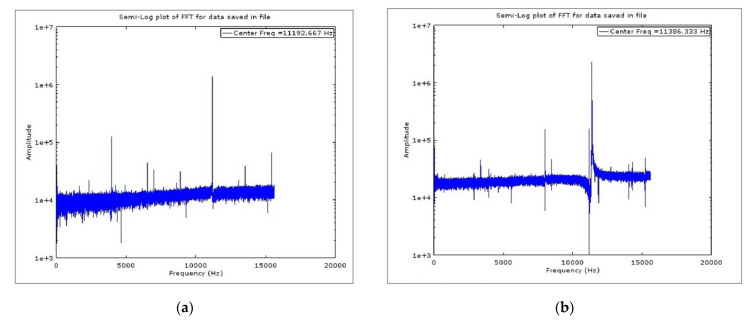
Fast Fourier Transformation for the dynamic response: (**a**) drive; (**b**) sense.

The dynamic response of BFVG is obtained by frequency scanning with the dynamic signal analyzer. The frequency sweep of drive mode is centered on 11 kHz, the span is 3.2 kHz and the level is 300 mVpk. At the same time, VPP is connected with 5 V DC voltage to obtain the output of the drive mode. The test procedure of the sense mode is similar to that of the drive mode. As shown in Figure 8, the drive frequency is 11,192 Hz and the sense frequency is 11,386 Hz. Table 2 shows the comparison of the simulation and experimental frequency results. The process error makes the actual drive and sense frequency of the gyroscope deviate by 8.989% and 5.367%, compared with the simulation.

### 4.2. Time Domain Dynamic Response

The Q factor is one of the most important performance parameters of the MEMS gyroscope, which reflects the energy storage capacity of a device. It is defined as the ratio of the energy stored in the device to the energy dissipated in each resonance period.

(1) $$Q=2\pi \frac{Energy_{stored\;in\;the\;resonator}}{Energy_{dissipated\;per\;cycle}}$$

For MEMS gyroscopes, the Q factor is mainly affected by air damping, anchor damping and thermoelastic damping (TED). Increasing the Q factor can effectively reduce the mechanical and thermal noise of the gyroscope. The total Q factor is the sum of the respective Q factors under the action of each factor.

(2) $$Q_{total}=\sum Q_{individual}$$

The gyroscope can be equivalent to a second-order system [12].

(3) $$m\ddot{x}+c\dot{x}+kx=0$$

m is the mass, k is the spring stiffness, and c is the damping factor. x(t) is the instantaneous displacement of the sensing structure from the equilibrium position.

By solving Equation (3), we obtain [12]:

(4) $$\omega =\sqrt{\frac{k}{m}}$$

(5) $$x\left(t\right)=Ae^{-\lambda t}\sin\left(\sqrt{\omega^{2}-\lambda^{2}t}+\varphi \right)$$

(6) $$\lambda =\frac{c}{2m}$$

A is the initial amplitude, 1/λ stands for the relaxation time τ . It can be seen from Equation (5) that the amplitude of vibration will decrease exponentially with time, which is the effect of damping. For an MEMS vibratory gyroscope with parallel plate capacitance design, air damping will greatly affect the energy dissipation of the structure, thereby affecting the Q factor [11,12].

(7) $$E=\frac{1}{2}kx^{2}$$

By substituting Equations (5) and (7) into the Equation (1), Q can be derived as shown in Equation (8) [12].

(8) $$Q=\pi \frac{1}{\lambda }f=\pi \tau f$$

In practical measurement, the Q factor is determined by the free vibration of the gyroscope in the time domain. Free vibration means that the driving force is turned off. At this time, the vibration amplitude will decrease exponentially with time. 

**Figure 9:**
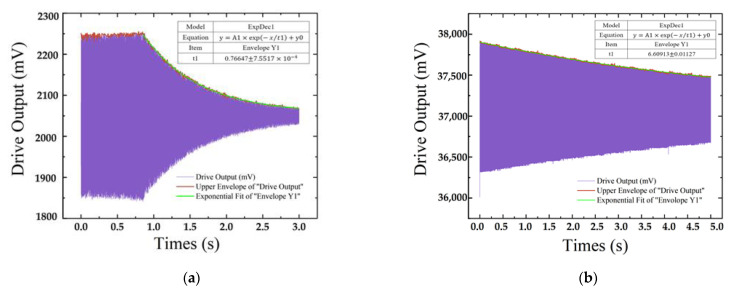
The time domain dynamic response measured at the drive mode of the gyroscope: (**a**) ceramic shell package; (**b**) novel 3D package.

Figure 9 shows the time domain dynamic response measured under the drive mode of the gyroscope. From the upper envelope (green) fitting results of Figure 9a,b, it can be seen that the relaxation times τ of ceramic shell packaging and novel 3D wafer-level packaging are 0.766 s and 6.609 s, respectively. The Q factor of the gyroscope at the drive frequency of 11,192 Hz can be calculated as 26,919 and 232,259, respectively, according to Equation (8).

**Figure 10:**
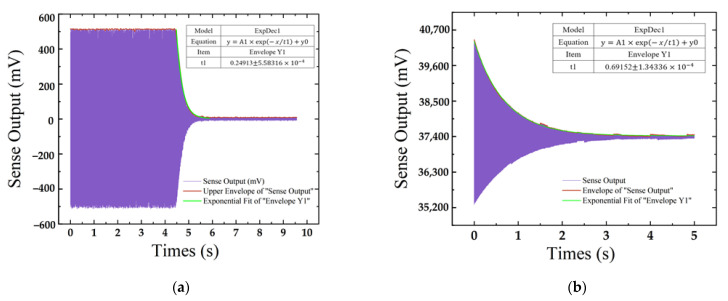
The time domain dynamic response measured in the sense mode of the gyroscope: (**a**) ceramic shell package; (**b**) novel 3D package.

Figure 10 shows the time domain dynamic response measured at the sense mode of the gyroscope. Figure 10a,b show that the relaxation time τ of the ceramic shell packaging and novel 3D wafer-level packaging are 0.249 s and 0.692 s, respectively. The Q factor of the gyroscope at the sense frequency of 11,386 Hz can be calculated as 8902 and 24,740, respectively, according to Equation (8).

As shown in Table 3, the Q factors of the BFVG drive and sense modes in relation to the novel 3D package are 8.628 times and 2.779 times higher than that in the traditional ceramic shell package, respectively.

The Q factors of three typical butterfly gyros processed in different ways are selected and compared with those of this group. The results are shown in Table 4. Nils Hedenstierna [8] obtained a high Q factor by processing glass–silicon–glass three-layer structure anodic bonding. Finally, the Q factors of drive mode and sense mode were 170,000 and 1600, respectively. Xu Qiang [21] manufactured a movable sense structure by bonding monocrystalline silicon and glass, and encapsulated it with a ceramic shell. The Q factors of the drive mode and sense mode were 14,296 and 4084, respectively. J. Su [4] obtained the Q factors of the drive mode and sense mode through a certain vacuum package, which were 16,830 and 1052, respectively.

It can be seen from Table 4 that the results of the new manufacturing technology are obviously better than those for the butterfly gyroscope reported previously. The ultra-high Q factor of BFVG shows that 3D wafer-level packaging technology can effectively facilitate the reliable packaging of gyroscopes.

### 4.3. Full-Scale Range and Nonlinearity

**Figure 11:**
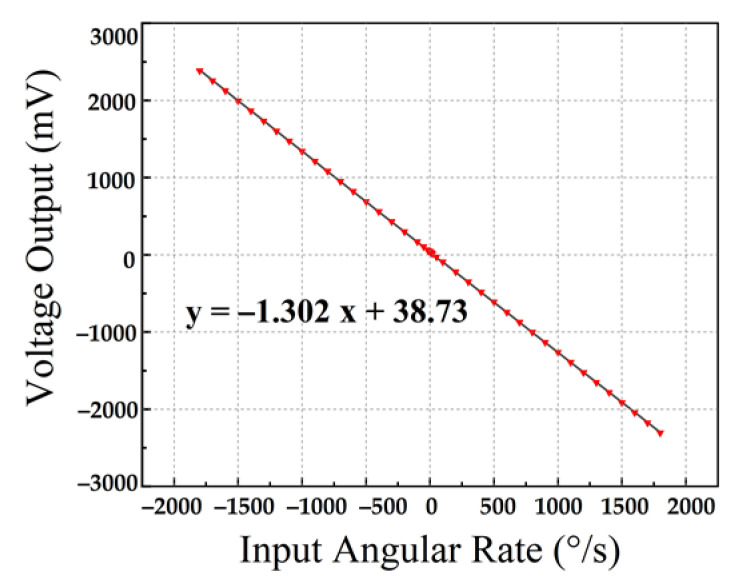
Full-scale testing results and nonlinearity of BFVG.

The scale factor refers to the ratio of the gyroscope output value to the input angular rate. Within the range of maximum input angular rate, all the measured data are obtained through dynamic experimental measurement. The slope of the straight line is obtained by fitting with the least square method. This is called the “scale factor”, also known as “scale factor linearity”. The residual error of linear slope fitting determines the credibility of the fitting data, which represents the deviation from the actual input and output of the gyro. We place the encapsulated gyroscope board on the angular velocity meter to test the angular velocity performance. When electrostatic excitation is applied to the drive element, multiple angular rates are applied on the *z*-axis. Figure 11 shows the test results. The processed gyroscope has a high resolution of 0.1°/s, a scale factor of 1.302 mV/(°/s), and nonlinearity of 558 ppm in the full-scale range of ±1800°/s.

### 4.4. Stability Test

**Figure 12:**
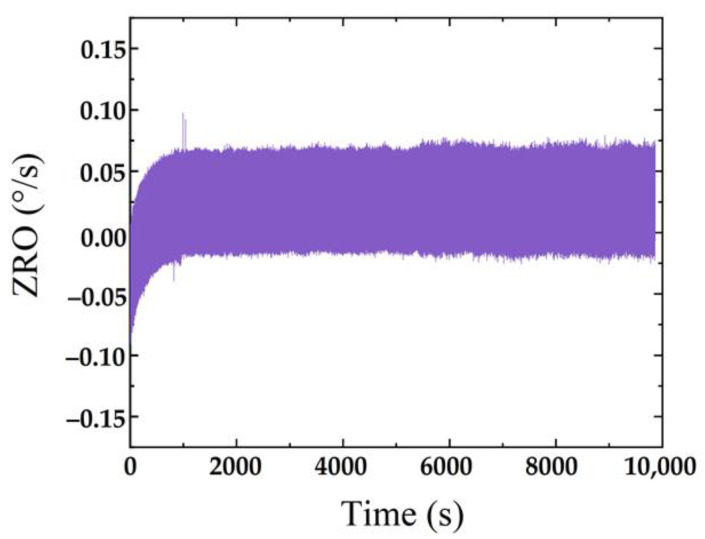
Room-temperature ZRO results.

Zero-rate output (ZRO) refers to the average value of the output angular rate measured in the state of zero input within the specified time. The ZRO characteristics at room temperature were measured by a sense mode circuit system. The output angular rate data were recorded at a sampling rate of 1 kHz for 3 h. As shown in Figure 12, the ZRO results show an obvious drift trend in the initial sampling stage. However, the drift gradually decreases with time and stabilizes at about 0.085°/s after about 15 min.

**Figure 13:**
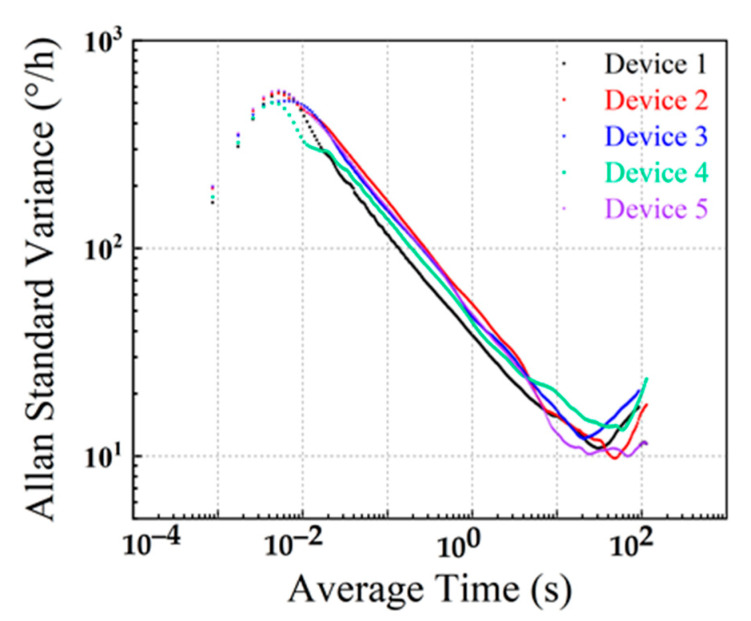
The Allan standard variance analysis results for typical BFVG.

Allan standard variance analysis was performed on the fitted ZRO results. The results are shown in Figure 13. The two important BFVG parameters of angular random walk (ARW) and bias instability (BI) are extracted from each Allan standard variance diagram. ARW refers to the gyro output error coefficient accumulated over time caused by white noise. It reflects the uncertainty (angular random error) of the angular velocity integral of the gyro output over time. Its physical definition is the standard deviation under the square root of the unit detection bandwidth. The BI of the gyroscope is used to measure the dispersion of gyroscope output around its mean value when the input angular rate is zero. It is expressed by the equivalent input angular rate corresponding to the standard deviation of the output within the specified time. The ARW and BI of five devices are listed in Table 5. The best performed device’s ARW and BI are 1.281°/√h and 9.789°/h, respectively. It can be seen from Figure 13 and Table 5 that the manufactured devices have a good consistency.

## 5. Conclusions

In this paper, a new 3D sandwich packaging technology composed of composite substrate–SOI–glass cap is developed and applied to the manufacture of an ultra-high Q butterfly MEMS gyroscope (BFVG). The composite substrate is processed by TSV and GIS to ensure a high vacuum degree and realize the vertical electrical interconnection by low-resistance silicon pillars. The BFVG was tested and characterized by self-developed circuits. The Q factors of drive and sense modes are 8 times and 2 times higher than those of traditional ceramic shell packaging, respectively. In addition, the BFVG achieves a high resolution and large measurement range, and small scale factor nonlinearity. The process error makes the actual drive and sense frequencies of the gyroscope deviate by 8.989% and 5.367% compared with the simulation. This novel ultra-high Q factor BFVG with 3D wafer-level sandwich packaging shows great potential to achieve higher performance.

## Figures and Tables

**Table 1:** Important structure parameters in the simulation.

Properties	Value	Unit
Thickness of structure	20	μm
Etching depth of asymmetric coupling beam	3	μm
Width of coupling beam	1	μm
Length of coupling beam	94	μm
Etching depth of asymmetric supporting beam	3	μm
Width of supporting beam	1	μm
Length of supporting beam	51	μm
Young’s modulus	190	GPa
Mass density	0.023	kg/cm^3^
Poisson’s ratio	0.266	null

**Table 2:** Comparison of simulation and experimental frequency results.

Mode	Simulation (Hz)	Experimental (Hz)	Error (%)
drive	10,269	11,192	8.989
sense	10,806	11,386	5.367

**Table 3:** Q-factor comparison between ceramic shell package and 3D wafer-level package.

Mode	Ceramic Shell Package	Novel 3D Wafer-Level Package	Improvement (Times)
drive	26,919	232,259	8.628
sense	8902	24,740	2.779

**Table 4:** The Q factor of BFVGs with different processing.

BFVGs	Manufacture Technology	Mode	Q Value
Nils Hedenstierna [8]	Glass–silicon–glass anodic bonding	drive	170,000
		sense	1600
Xu Qiang [21]	Ceramic shell package	drive	14,296
		sense	4084
J. Su [4]	Certain vacuum package	drive	16,830
		sense	8052

**Table 5:** The Allan standard variance results of five typical BFVGs.

Devices	ARW (°/√h)	BI (°/h)
device 1	1.358	10.534
device 2	1.586	9.789
device 3	1.649	12.242
device 4	1.824	13.621
device 5	1.281	10.061
